# Current Therapies and New Targets to Fight Melanoma: A Promising Role for the β3-Adrenoreceptor

**DOI:** 10.3390/cancers12061415

**Published:** 2020-05-30

**Authors:** Luca Filippi, Gennaro Bruno, Vladana Domazetovic, Claudio Favre, Maura Calvani

**Affiliations:** 1 Neonatal Intensive Care Unit, Medical Surgical Feto-Neonatal Department, A. Meyer University Children’s Hospital, 50139 Florence, Italy; 2 Department of Health Science, University of Florence, 50139 Florence, Italy; vladana.domazetovic@unifi.it; 3 Department of Paediatric Haematology-Oncology, A. Meyer University Children’s Hospital, 50139 Florence, Italy; claudio.favre@meyer.it (C.F.); maura.calvani@meyer.it (M.C.)

**Keywords:** melanoma, β3-adrenergic receptor, cancer therapies

## Abstract

Melanoma is one of the most aggressive types of cancer and the most deadly skin cancer. According to World Health Organization, about 132,000 melanoma skin cancers occur globally each year. Thanks to the efficacy of new therapies, life expectation has been improved over the last years. However, some malignant melanomas still remain unresponsive to these therapies. The β-adrenergic system, among its many physiological roles, has been recognized as the main mediator of stress-related tumorigenic events. In particular, catecholamine activation of β-adrenergic receptors (β-ARs) affects several processes that sustain cancer progression. Among the β-AR subtypes, the β3-AR is emerging as an important regulator of tumorigenesis. In this review, we summarize data of different experimental studies focused on β3-AR involvement in tumor development in various types of cancer and, particularly, in melanoma. Taken together, the preclinical evidences reported in this review demonstrate the crucial role of β3-AR in regulating the complex signaling network driving melanoma progression. Therefore, a need exists to further disseminate this new concept and to investigate more deeply the role of β3-AR as a possible therapeutic target for counteracting melanoma progression at clinical level.

## 1. Introduction

Melanoma is still one of the most aggressive and chemotherapy-resistant human cancers, originating from melanocytes. Melanoma development, as for the majority of skin cancers, is related, at least in part, to ultraviolet radiation exposure and to years of any protracted sun/UV exposure. This relationship explains why melanoma location is related to sun exposure [1]. Further risk factors for melanoma development include a particularly fair skin, hair, and eye color phenotypes associated with increased sun sensitivity, the presence of dysplastic nevi, and genetic predisposition [2].

The incidence of invasive melanoma has been rising worldwide over the past two decades, despite numerous efforts to enhance primary prevention and early detection [3]. The overall incidence has been increasing particularly in the United States, with an increase by 270% over the past 30 years [4]. Melanoma predominantly affects the Caucasian population, whose risk is 10-fold increased compared to people with dark skin pigmentation [4], and represents the fifth most frequent cancer in men and the sixth most common cancer in women in the United States [5]. The region with the highest incidence of melanoma is Australia with the age-standardized incidence rate increased by 181% between 1982 and 2016, from 27 cases per 100,000 in 1982 to an estimated 50 cases per 100,000 in 2016 [6]. 

It has been assessed so far that adrenergic system, via catecholamine release following stress events, is able to sustain cancer progression and influence the outcome of patients affected by several malignancies. In this contest, β-adrenergic receptors (β-ARs) have been identified as the main responsible actors of stress-enhanced tumor-related pathways [7]. β-ARs are G protein-coupled receptors responsible for mediating many physiological and pathological responses in humans and animals. Although initially two β-AR subtypes have been identified, β1-AR and β2-AR [8], the existence of the third β-AR subtype, firstly described as “atypical β-adrenoreceptor” [9], and named β3-AR, has been acknowledged much later. We know that all three β-ARs can be activated by noradrenaline and adrenaline, although with different affinity. The two catecholamines are, indeed, equipotent at the β1-AR, while adrenalin is 100-fold more selective for β2-AR. On the contrary, noradrenaline is more potent than adrenaline as a β3-AR agonist [10]. All three β-ARs are expressed in numerous cell types of the tumor microenvironment; therefore, dissecting the effects related to each specific β-AR subtype involved in tumor progression is a complex but required objective.

Since in melanoma, especially in resistant subtypes, new therapies are needed; in light of a continuous search for alternative therapeutic strategies, β-AR modulators cannot be forgotten. In particular, recent preclinical studies evidence the emerging role of β3-AR in melanoma.

## 2. Current Therapies in Melanoma

For cutaneous melanomas localized and not outspread beyond the site of origin, surgical excision remains the primary modality of treatment, with a high five-year relative survival rate but yet dependent on the tumor mass thickness and ulceration. However, survival percentage drops dramatically for patients diagnosed with advanced or metastatic melanoma (65% for regional to 25% for distant melanoma lesions), because treatment options are limited [11]. Chemotherapy, such as Dacarbazine (DTIC) [12] or Temozolomide [13], has been shown to offer only modest benefits and severe adverse effects, and has not been able to significantly change the prognosis. However, in the past decade, the approval of many new treatments for metastatic disease has contributed significantly to the recent increase in survival rate, as recently reported by the American Cancer Society [14].

In 2011, the US Food and Drug Administration (FDA) approved Ipilimumab for metastatic disease therapy, the first immune checkpoint inhibitor (an anti-CTLA-4 drug), for the treatment of advanced melanoma, and Vemurafenib, a BRAF inhibitor, for the treatment of unresectable or metastatic melanoma with BRAF V600E mutation. Then, two new monoclonal antibodies, Nivolumab and Pembrolizumab targeting the anti-programmed cell death protein 1 (PD-1) were approved and marketed in 2014 [15]. At the same time, another drug able to inhibit BRAF, Dabrafenib, was approved by the FDA in 2013 [16], but also MEK inhibitors, drugs able to inhibit the mitogen-activated protein kinase enzymes MEK1 and/or MEK2, such as Trametinib or Cobimetinib, were made available and approved, respectively, in 2013 and 2015. All these new drugs have been used alone or in combination [17]. These new pharmaceutical opportunities, and the ability to combine these drugs to target different immune checkpoints, have contributed to the sharp drop in mortality [18]. The overall survival rate at 5 years was 52% in patients treated with Nivolumab and Ipiliumubab versus 44% in patients treated only with Nivolumab and 26% in those treated only with Ipilimumab [19]. Alongside the higher cost of biological therapies compared to traditional ones for the treatment of melanoma, the significant increase in survival has been associated with an impressive increase of expenses, raising the question of financial sustainability, mainly for public health systems [20]. For this reason, the availability of new options, hopefully less expensive, represents a matter of priority. 

Despite the significant improvements in life expectations for patients with historically poor outcomes attributable to new immunotherapies, a significant number of patients still remain far from significant long-lasting benefits. Therefore, alternative therapies are definitely required.

## 3. Role of Stress and β-ARs in Cancer

The longstanding hypothesis of the existence of a relationship between stress (for example related to surgery, but also linked to psychosocial factors, such as depression) and tumor onset, has been confirmed in the last years through numerous experimental and clinical investigations [21,22]. The explanation of this relationship must be sought in the activation of the sympathetic nervous system and the release of stress-related mediators such as catecholamines, whose concentrations increase in the tumor microenvironment [23]. In particular, sympathetic nervous system and catecholamine exert pro-tumorigenic actions through the modulation of β-ARs, which in turn affect several biological processes related to cancer progression or metastasis, such as tumor cell proliferation, invasiveness, migration and vascularization [7,22,24,25]. Psychological aspects affecting melanoma patients, such as fear of cancer recurrence, anxiety, depression and risk of suicide, have been documented by several studies [26,27,28]. Therefore, in light of the existing link between stress and cancer progression in melanoma patients, interventions aimed to provide efficient psychological support are of uttermost importance in management of melanoma patients [29]. 

In preclinical murine models, β-ARs antagonists have shown the ability to block stress-induced enhancement of tumor progression in several malignancies, including melanoma [30,31], breast [32], prostate [33], and leukemia [34] cancers. On the contrary, β-ARs agonists have been found to enhance in vivo tumor progression and metastasis [32,33,35], confirming the critical involvement of β-ARs in cancer biology. Among the direct effects exerted on tumor cells, some reports demonstrated that β-ARs are also able to regulate pathways in non-tumoral cells of tumor microenvironment [36]. Despite the first data highlighted a prominent role of the β2-AR subtype in regulating tumor-relating pathways [37], recent studies have directed attention to β3-AR subtype as an important player in cancer biology. 

## 4. β-ARs in Melanoma

The involvement of stress in melanoma development and progression is definitely clear and well established by biological and clinical evidences [38]. A large amount of in vitro and in vivo experimental data together with epidemiological studies have shown that β2-AR is the β-AR subtype mostly involved in mediating the effects of catecholamines in cancer [39].

In melanoma, the role of β-adrenergic system has been evaluated by in vitro and in vivo studies before testing β-adrenergic blockers in humans. In vitro studies confirmed the presence of β-ARs in animal and human melanoma cells. These studies also confirmed the role of β-ARs in the modulation of angiogenesis and demonstrated that non-selective β-AR antagonists, but not β1-AR selective antagonists, promote apoptosis of cancer cells [40,41,42]. Propranolol exerts potent anti-tumoral effects, attenuating migration, reducing vascular endothelial growth factor (VEGF) secretion and inducing apoptosis in both cutaneous and uveal melanoma in a dose-dependent manner [43]. Anti-angiogenic effect of propranolol has also been well described in infantile hemangiomas (IHs) and retinopathy of prematurity [39]. In fact, a significant reduction of serum VEGF levels has been demonstrated in infants affected by IHs after one or two months of propranolol treatment [44,45]. A similar effect has been demonstrated in hypoxic retina [46]. Accordingly, VEGF expression is down-regulated by propranolol in a dose-dependent manner in hemangioma-derived endothelial cells [47] and in hemangioma-derived stem cells [48]. All these data suggest that the anti-tumor activity of propranolol relies in part on its ability to block tumor angiogenesis. Besides its anti-angiogenic activity, propranolol affects activities of other cells in tumor microenvironment. In ovarian carcinoma, propranolol decreases macrophage recruitment by tumor mass [49], and in a model of breast cancer it prevents macrophage M2 polarization [50]. In melanoma, propranolol decreases the infiltration of immunosuppressive myeloid cells, such as neutrophils, in the tumor microenvironment and favors the cytotoxic tumor-infiltrating lymphocytes’ activity [51]. Moreover, the pathogenic role of stress in promoting melanoma growth in human cells and in mice was described by the efficacy of propranolol used alone [30,42,52] or associated with other drugs [53]. 

Of particular interest is the recent demonstration of the existing relationship between stress and immune depression, which is indicated as a decisive factor for tumor progression and metastasis development [54,55,56]. The β-adrenergic system has been identified as one of the major players in the regulation of the immune system. In particular, it was demonstrated that increased catecholamine levels induced both suppression of natural killer (NK) cell cytotoxicity [57,58,59], leading to tumor metastasis [34,60], and reduction of cytolytic killing ability of antigen-specific CD8^+^ T cells [61]. Moreover, β-adrenergic system promoted an increase of T regulatory (Treg) cell suppressive activity, through β2-AR signaling [62] and an accumulation of myeloid-derived suppressor cells (MDSC) in mice [63] and in human patients [64]. These effects were mediated by an increased production of norepinephrine [65]. Interestingly, a preclinical study demonstrated that in mice bearing B16-F10 melanoma cells, treatment with propranolol was able to potentiate immune-based therapies [66]. These preclinical evidences suggested to better explore the possible efficacy of β-blockers as anti-cancer adjunctive treatment [51,67] and to improve the efficacy of cancer immunotherapy [68].

The strongest evidence of the role played by β-ARs in regulating distinct aspects of melanoma cancer comes from clinical investigations showing the efficacy of non-selective β-ARs’ antagonists in reducing the malignancy progression. Numerous observational studies suggested that patients treated initially with β-ARs blockers for cardiovascular diseases and hypertension have also shown reduced melanoma risk [69,70,71]. De Giorgi et al. demonstrated that patients with thick malignant melanoma (thickness > 1 mm) and concomitantly treated with β-blockers for one year or more, were associated with a reduced risk of tumor progression. In fact, the risk of disease progression (assessed by the presence of sentinel lymph node metastases and lymphatic, in-transit, or visceral metastases) was significantly reduced by 36% for each year of β-blockers use. Although the number of patients included in this study was relatively small a significant reduction in mortality was observed in patients treated with β-blockers [69]. Clinical follow-up of these patients after eight years from treatment with β-blockers confirmed the reduced disease progression from 45% to 30%, and reduced mortality from 35% to 17% [70]. The study of Lemeshow et al. supports a similar conclusion: a large population-based cohort study showed that the exposure to β-blockers for more than 90 days prior to diagnosis induced a lower mortality due to melanoma compared to the no-exposure group [71]. However, two large epidemiologic studies performed in patients who received β-AR antagonists (the majority assuming β1-AR selective blockers) did not confirm these results, even though the risk improved with larger amounts of cumulative daily dose [72,73]. A clinicopathological study evaluated the prognostic significance of β2-AR expression on surgically resected cutaneous malignant melanoma. This study revealed that β2-AR expression was positively correlated with tumor thickness, ulceration, disease stage, and finally with a poor overall survival [74]. A recent study observed a significant survival benefit for patients treated with immunotherapy and taking unselective β-blockers compared to either those receiving no β-blockers or β1 selective-blockers [66]. Moreover, a more recent prospective study performed in patients with cutaneous melanoma demonstrated that propranolol treatment reduced the risk of recurrence by 80% [75].

## 5. β3-AR and Cancer

The first report, showing that β3-AR influences the risk of cancer, suggested that a polymorphism in codon 64 of the β3-AR gene, that features replacement of tryptophan by arginine (Trp64Arg), decreased the risk of breast cancer in Japanese women. In particular, individuals who simultaneously carried a glutamic acid polymorphism in β2-AR gene (Gln27Glu) together with the Trp64Arg β3-AR polymorphism had the most markedly reduced risk of breast cancer, with an odds ratio of 0.37 [76]. In a second study, the same β3-AR polymorphism (Trp64Arg) was associated with susceptibility to endometrial cancers in overweight/obese individuals [77]. From these first studies, an involvement of β3-AR in the biology of cancer was beginning to emerge. However, until few years ago, the poor knowledge regarding the β3-AR distribution and pharmacology, and the lack of selective tools suitable for the study of this β-AR subtype, has made difficult to clarify its contribution in the complex landscape of tumor biology. Indeed, the presence of β3-AR was first established in physiological tissue, primary in brown adipocytes where it mediates thermogenesis [9], and, for many years, its expression in tumor tissues was completely unknown.

For the first time, in 2008, a significant up-regulation of the β3-AR mRNA was described in a tumor tissue; a study involving 41 patients affected by colorectal cancer suggested a possible involvement of the β3-AR in the pathogenesis of this malignancy [78].

In 2013, in a very interesting study, Magnon et al. [79] investigated the role of adrenergic signals in prostate cancer development. To assess the contribute of the β-ARs, human prostate PC-3 cancer cell line was injected in the prostate of mice genetically deficient for β2-, β3-, or both β2- and β3-ARs. The first relevant observation was that tumor growth in the prostate was slightly delayed when mice were lacking β2- or β3-AR singularly, but it was severely compromised in *ADRB2−/−ADRB3−/−* mice. Notably, in the double knockout mice, prostate cancer cell dissemination into the lymph nodes and other distant organs was significantly reduced. These results were also confirmed by using the human prostate LNCaP cell line in the same animal model, suggesting that both β2- and β3-ARs, expressed in stromal cells of the tumor microenvironment, are critically involved in tumor development and metastatic dissemination of this malignancy.

Recently, β3-AR mRNA and protein expression have been reported across different tumors including vascular tumors, breast cancers and human leukemia cells [80,81,82]. Notably, in these diseases, β3-AR mRNA or protein expression were strongly increased compared to the healthy counterpart tissues. Moreover, new evidence on β3-AR expression was obtained in many other tumors [83], confirming the hypothesis that this β-AR subtype could play a pivotal role in the onset and/or progression of numerous malignancies [Table 1]. Accordingly, a β3-AR gene variant has been found implicated in the predisposition to gallbladder cancer, the most common and highly aggressive biliary tract malignancy [84]. In addition to several studies on melanoma, discussed below, we recently demonstrated that β3-AR is expressed in both murine and human neuroblastoma (NB) cell lines, and in tumor biopsies from NB patients; in this study, pharmacological antagonism of β3-AR, in a murine syngeneic model of NB, was able to reduce tumor growth by affecting the neuronal differentiation of NB cancer cells [85].

## 6. β3-AR in Melanoma: Preclinical Studies

A prospective cohort study has demonstrated that the most used β-ARs antagonist, propranolol, an approved drug with a non-oncology primary purpose, protected patients with thick cutaneous melanoma from melanoma recurrence [75]. Propranolol is reported to have an order of affinity for β2-, β1- and β3-AR respectively of 0.8, 1.8, and 186 nM [93,94]. The protective effects observed in melanoma patients treated with propranolol suggested, therefore, a clear involvement of the β2-AR subtype in melanoma progression. However, it should be emphasized that an involvement of the other β-AR subtypes in the observed effects related to melanoma progression cannot be excluded, owing to the broad dosage spectrum with which propranolol and other β-ARs antagonists have been used in clinical practice [95], and considering that a clear-cut selectivity remains questionable for most β-blockers [96]. Unfortunately, due to the lack of clinical studies using selective β3-AR antagonists in humans, the role of β3-AR subtype in melanoma cancer has not been clarified so far at clinical level; nevertheless, its contribution to processes related to melanoma progression is becoming evident, as suggested by pre-clinical evidences.

Studies on murine B16-F10 melanoma cells demonstrated for the first time that the pharmacological β3-AR blockade was able to reduce proliferation and induce apoptosis of melanoma cells in vitro; these effects were also reproduced by using a siRNA molecular approach targeting specific β-ARs [86]. In the same study, thorough a murine syngeneic experimental model (B16-F10 inoculated in C57BL mice), β3-AR antagonists SR59230A and L-748,377, were both able to significantly reduce melanoma growth in vivo, in comparison with propranolol, by reducing cell proliferation and inducing apoptosis of cancer cells. In addition, the β3-AR blockade was also able to reduce tumor vasculature through apoptosis of endothelial cells [87]. The effects of β3-AR modulation on melanoma cell proliferation and survival were found mediated by the inducible nitric oxide synthase (iNOS) demonstrating that iNOS-produced nitric oxide (NO) is a downstream effector of β3-AR signaling in melanoma [88]. Notably, melanoma cell proliferation was inhibited by β3-AR blockade either in the presence or not of noradrenaline stimulation, indicating that a partial constitutive β3-AR activity, already hypothesized [97], was present in melanoma cells and may contribute to the proliferation process [86].

**Figure 1:**
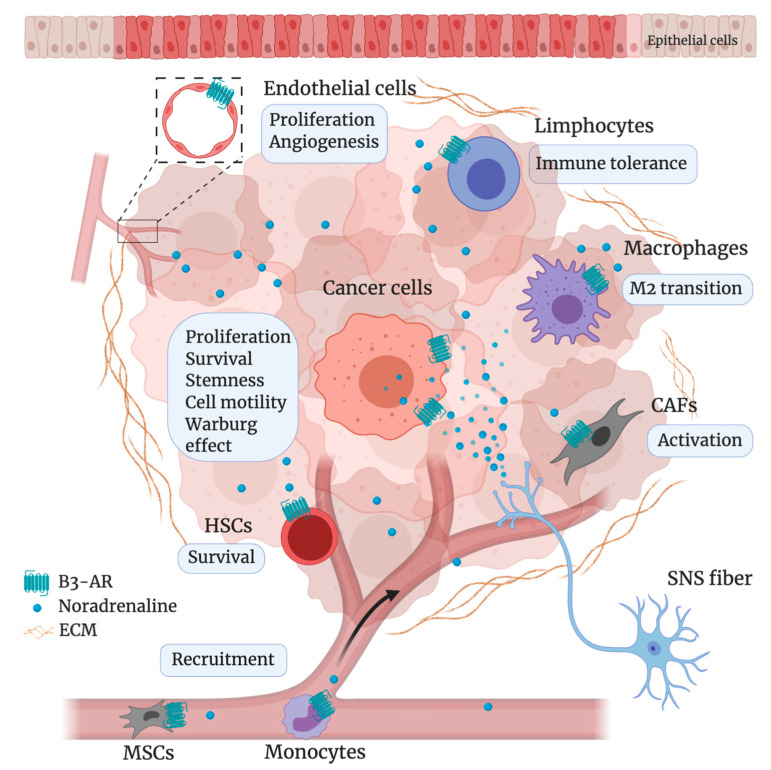
Schematic representation of β3-AR-regulated processes in tumor and stromal cells of melanoma microenvironment (TME). A complex network of interaction and crosstalk between tumor and stromal cells of the TME, sustained through β3-AR-enanched processes, promotes melanoma progression. (CAFs = cancer-associated fibroblast; ECM = extracellular matrix; HSCs = hematopoietic stem cells; M2 = macrophage type 2; MSCs = mesenchymal stem cells; SNS = sympathetic nervous system). Created with BioRender.

Both β2- and β3-AR were found strongly expressed and actively functional in different stromal cells of the TME, such as cancer-associated fibroblasts (CAF), macrophages and endothelial cells [89]. Indeed, besides the direct effects elicited by the β3-AR modulation in melanoma cells, several studies have shown how this receptor is able to regulate stromal, inflammatory, vascular and immune cells of the melanoma microenvironment [87,89,90], thus contributing to the regulation of numerous processes related to melanoma malignancy. In particular, β3-AR is able to elicit stromal reactivity, sustain secretion of pro-inflammatory cytokines and drive *de novo* angio/vasculogenesis [89]; the same study has confirmed that β3-AR instructs melanoma cells to respond to environmental cell signals and to sense CAFs and macrophages enhancing their tumorigenic and stem-like traits. In regard to the immune regulation, pharmacological and molecular approaches with β-blockers (propranolol and SR59230A) and specific siRNA targeting of β2- or β3-ARs injected in B16-F10 melanoma-bearing mice, suggested an involvement of β3-AR subtype in the regulation of the immune-tolerance in melanoma microenvironment [90]. Indeed, β3-AR blockade increased the number of NK cells and lymphocytes CD8^+^ as well as their cytotoxicity, M1/M2 macrophages ratio and N1 granulocytes, while it abrogated Treg and MDSC sub-populations in tumor mass. By reducing the immune-suppressive and increasing the immune-competent subpopulations of cells in the TME, the β3-AR blockade proved the hypothesis that β3-ARs might play a role in the promotion of immune tolerance of melanoma. Taken together, these data confirm the pivotal role played by the β3-AR in regulating several biological processes related to melanoma progression (Figure 1).

Recently, it has been demonstrated that in murine B16-F10 melanoma-bearing mice, the pharmacological β3-AR blockade was able to reduce the expression of cancer stem cell (CSC) markers, and to induce a differentiated phenotype of numerous hematopoietic progenitors recruited in TME [91]. The differentiation of melanoma and various stromal cells involved in pro-tumorigenic processes, brought about by the β3-AR blockade at the expense of stemness traits, thus hitting the metastatic potential of melanoma, could represent an efficacious strategy to counteract the progression to advanced stages of this malignancy.

In human A375 melanoma cells, β3-AR stimulation through the selective agonist BRL37344 was able to induce a shift from an oxidative to a glycolytic metabolism, sustaining a metabolic process typical of tumor cells and known as Warburg effect [92]. Notably, β3-AR expression was found increased in melanospheres of A375 melanoma cells compared to the parental cell line, once again confirming that the β3-AR expression may correlate with pathways related to stemness features of tumor cells. β3-AR activation, indeed, induced the expression of specific glycolytic enzymes, such as hexokinase 2 (HKII), monocarboxylate transporter-4 (MCT-4), and glucose transporter-1 (Glut-1), which reflected elevated glucose uptake and lactate overproduction, two key metabolites of the Warburg effect. Moreover, the β3-AR/UCP2 axis strongly affected the mitochondrial activity by reducing ATP synthesis and mitochondrial reactive oxygen species (mtROS) content in melanoma cells. All these effects were reverted by using the β3-AR antagonist SR59230A, highlighting the crucial role played by the β3-AR in regulating molecular signaling that sustain metabolic and energetic processes typical of cancer stem cells [92].

Hypoxic induction of the β3-AR protein has been reported in murine B16-F10 and human A375 melanoma cells [86,89]. Hypoxia is a well-known condition of solid tumors, including melanoma, able to orchestrate at cellular level a complex program, which leads to pro-tumorigenic events [98,99]. The induction of β3-AR expression in a hypoxic microenvironment could suggest that tumor cells exploit the activation of β3-AR pathways to achieve aggressiveness features required in the tumorigenic process.

Despite studies investigating the role of β3-AR at clinical level still not being available, the expression of this receptor has been confirmed in melanoma biopsies from different patients. An immuno-histochemical analysis for the expression of β3-AR has been assessed in different cutaneous human melanocytic lesions including common and atypical nevi, in situ primary melanoma, superficial spreading melanoma, nodular melanoma, cutaneous, and lymph-nodal metastatic melanoma. Although β3-AR was expressed in all examined melanocytic lesions, its expression level, taking into account both staining intensity and percentage of positive cells, was significantly higher in malignant compared to benign lesions [89]. Importantly, in these biopsies β3-AR was found expressed also in stromal, endothelial and inflammatory cells of the TME, in accordance with the data obtained at preclinical level. These data clearly suggested that β3-AR expression correlates with melanoma malignancy features in human melanocytic lesions.

## 7. Conclusions

Therapeutic options for patients with advanced-stage melanoma have been increased in the last years, and approval of new therapeutic agents such as the checkpoint inhibitors (anti-CTLA4 and anti-PD1 antibodies) and BRAF/MEK inhibitors has opened new expectations for survival. Moreover, early clinical data in a small patient population suggests that targeted therapy with BRAF/MEK inhibitors may work in synergy with checkpoint inhibitors and this triplet therapy may improve survival in patients with metastatic melanoma [100]. Despite the therapeutic improvement, some melanomas still remain unresponsive. Therefore, the discovery of new therapeutic strategies, especially for advanced melanoma, is still necessary not only to improve the cure rate but also the quality of life of these patients.

During the years, several studies have accumulated evidence that, in melanoma, both tumor cells and non-tumor stromal cells cooperate in a complex signaling network to sustain tumor growth and progression. This meticulously orchestrated arrangement often underlies the onset of resistance mechanisms or the recurrence of the disease in some therapeutic regimens, especially in those that hit targets involved in a single pathway of the entire network. Accordingly, identifying a therapeutic target involved in regulation of multiple pro-tumor signaling pathways could represent a successful approach.

In light of this, preclinical data summarized in this review have clearly suggested that β3-AR is able to modulate the activity of different cells in the melanoma microenvironment and, consequently, its blockade exerts an important anti-tumor action by affecting multiple pro-tumor signaling pathways. Even though further investigations are needed, especially at clinical level, these first experimental evidences highlight the functional role of the β3-AR subtype in melanoma malignancy, and suggest β3-AR as a therapeutically valid target to counteract melanoma progression.

## Figures and Tables

**Table 1:** Studies Describing β3-AR Involvement in the Development of Different Cancers.

β3-AR (mRNA, Protein, Gene) Evaluation	Species	Experimental Model	Disease	Biological or Clinical Observations	Study Type	Reference
Trp64Arg polymorphism	human	tissue sample	breast cancer	decreased risk of breast cancer	Case-control study	Huang XE et al., 2001 [76]
Trp64Arg polymorphism	human	tissue sample	endometrial cancer	susceptibility to endometrial cancers	Case-control study	Babol K et al., 2004 [77]
mRNA expression	human	tissue sample	colorectal cancer	overexpression related to neoplastic transformation	Case series study	Perrone MG et al., 2008 [78]
gene knockout	human	PC-3 cells and LNCaP cells in xenograft nude mouse model	prostate cancer	genetic deletion of stromal β2/3-ARs prevents the early phases of tumor development and tumor cell dissemination	Preclinical study	Magnon C et al., 2013 [79]
protein expression	human	tissue sample	infantile haemangioma	strong expression in the (early) proliferative phase	Descriptive study	Chisholm KM et al., 2012 [80]
protein expression	human	tissue sample	breast cancer	overexpression compared to normal breast tissue	Retrospective cross-sectional study	Montoya A et al., 2017 [81]
mRNA expression	human	Nalm-6 pre-B ALL cells in xenograft mouse model	acute lymphoblastic leukaemia	increased cell proliferation	Preclinical study	Lamkin DM et al., 2012 [82]
protein expression	human	tissue sample	different types of solid cancer	overexpression compared to normal tissues	Case-control study	Rains SL et al., 2017 [83]
rs4994 polymorphism	human	tissue sample	gallbladder cancer	increased risk of gallbladder cancer	Case-control study	Rai R et al., 2015 [84]
protein expression and modulation	murine, human	Neuro2A murine cells in syngeneic mouse model, SK-N-BE and BE(2)C human cells; tissue sample	neuroblastoma	β3-AR sustains tumor growth and proliferation, promotes stemness and blocks differentiation of NB cells	Preclinical study	Bruno G et al., 2020 [85]
protein expression and modulation	murine	B16-F10 syngeneic mouse model	melanoma	β3-AR blockade reduces tumor cell growth and proliferation, and tumor vascularization	Preclinical study	Dal Monte M et al., 2013 [86]
mRNA and protein expression; protein modulation	murine	B16-F10 syngeneic mouse model	melanoma	β3-AR promotes and sustains melanoma growth in concert with β1/2-ARs	Preclinical study	Sereni F et al., 2015 [87]
protein modulation	murine	B16-F10 cell model	melanoma	iNOS-produced NOis a downstream effector of β3-AR in melanoma	Preclinical study	Dal Monte M et al., 2014 [88]
protein expression and modulation	human	A375 cells; tissue sample	melanoma	β3-AR correlates with melanoma aggressiveness; β3-AR is involved in recruitment of circulating pre-stromal cells by the tumor, and enhances their pro-tumorigenic activity	Preclinical study	Calvani M et al., 2015 [89]
protein expression and modulation	murine	B16-F10 cell line; B16-F10 syngeneic mouse model	melanoma	β3-AR promotes immune tolerance in melanoma	Preclinical study	Calvani M et al., 2019 [90]
protein expression and modulation	murine	B16-F10 syngeneic mouse model	melanoma	β3-AR blockade induces differentiation of hematopoietic progenitors in TME	Preclinical study	Calvani M et al.,2020 [91]
protein expression and modulation	human	A375 cells	melanoma	β3-AR sustains metabolic and energetic processes in cancer cells (Warburg effect)	Preclinical study	Calvani M et al., 2018 [92]

AR = adrenergic receptor; iNOS = inducible NO synthase; NB = neuroblastoma; NO = nitric oxide; TME = tumor microenvironment.
